# Management of the Eye, Ear, and Throat

**Published:** 1887-01-08

**Authors:** 


### MANAGEMENT OF THE EYE, EAR, AND THROAT.

*The Eye and its Sight.*

All scientific works may perhaps claim to be instructive, but enjoyable, all certainly are not; it is, therefore, with peculiar satisfaction we recommend Mr. Power's little treatise to those interested in the eye. Mr. Power has been singularly successful in imparting a wide and vivid interest to his subject—a virtue by no means too common in medical writers, and when met with deserving all the more praise. The scope of the work is of course limited. It is in no sense a treatise on the diseases of the eye and their treatment; but is intended to serve, perhaps, a more important purpose—viz., that of teaching the reader the proper management of the eyes in the various stages and conditions of life, so that disease may be prevented, or at least modified. The anatomy and physiology of the eye, the effects of variations of light and temperature, colour blindness, squinting, effects of alcohol and tobacco, etc., are all treated in a masterly manner; Mr. Power being particularly happy in his choice of cases illustrating the various affections. Perhaps the most important chapter in the book is that treating of the causes and prevention of myopia or short-sightedness, an affection of great and increasing frequency. What with our school boards and competitive examinations, there seems to be considerable danger of the English nation becoming in a few generations as short-sighted and as learned as the German—an evil of no small magnitude. All those desirous of averting such a calamity would do well to study Mr. Power's remarks upon the subject, paying particular attention to the admirable instructions for the prevention of myopia.

*The Ear and Hearing.*

There are few organs in the human body more complicated in anatomy and physiology than the ear; none demanding clearer methods of description. Such being admittedly the case, in a semi-popular treatise like this of Mr. Field's, we expected difficulties to be smoothed and descriptions simplified as much as possible. This does not seem to be Mr. Field's opinion; or, perhaps, his extensive knowledge of the subject has somewhat blinded him to its difficulties and intricacies. The first part, treating of the anatomy and physiology of the ear, is very unsatisfactory. There is no regularity in description, no definite sub-division of the subject. The middle ear, Mr. Field describes as "a small air space with hard bony surroundings"; he makes no mention of its shape, says nothing about its walls. He confuses the reader by speaking at different places of the "tympanum," the "drum," the "drum cavity," the "hollow of the drum." Surely an unnecessary multiplicity of terms. The description of the inner ear or labyrinth is also complicated and unsatisfactory. Mr. Field is here candid enough to "recommend a more than cursory perusal of the minute structure of the inner ear only to those who are stout of heart." His head must be more than usually stout, who, entering for the first time the description of the labyrinth, emerges from it with his mental and moral faculties unimpaired. Following the anatomy and physiology, we have the more practical part of the treatise, in which "the causes of deafness," "injuries of the ear," "cerumen," "ear drops," "otorrhœa," etc., are discussed. This part is more satisfactory, and contains a great deal of useful and interesting information. But, there is here also great want of arrangement; there is no attempt whatever made to classify the information. It reminds one of the Scotch dish "haggis," composed of excellent material, but, from the mixture of so many ingredients, somewhat difficult of digestion. To the aurist the work will of course prove simple; to the ordinary practitioner it will be somewhat confusing, and of little practical use, while to the general reader the perusal of it will, we fear, result only in "confusion worse confounded." The subject is undoubtedly difficult, more undoubtedly dry; but, from a man of Mr. Field's abilities, we expected a work more simple and more interesting. Mr. Field is probably the ablest aurist of the day, as well as a noble-hearted man, and we make no doubt the defects in form we have felt bound to notice will disappear altogether in a second edition of his book.

*The Throat, with Voice and Speech.*

From the pen of Dr. Bristowe we always look for interesting matter, well arranged and ably written. We are pleased to find the little work before us in no way disappoint our expectations, the subject being treated in a simple, interesting, yet thoroughly scientific manner; the outcome of accurate knowledge and philosophic reasoning. Dr. Bristowe, wisely pre-supposing entire ignorance of the subject on the part of his readers, prefaces his remarks on "Voice and Speech" by a short sketch of the acoustics concerned in their production, the perusal of which will materially aid the reader in understanding the somewhat difficult subject which follows. The voice instrument, or larynx, has resemblance to three varieties of musical instruments, viz., the organ, stringed instruments, and reed instruments. These three varieties are shortly described by Dr. Bristowe, their points of resemblance and points of difference to the larynx being discussed. Dr. Bristowe concluding by suggesting, we think correctly, that the larynx may be best described as a reed instrument, but one "which in the main obeys the laws of strings, and which determines the notes of the voice in accordance with these laws." The nature of vowels and consonants, and the manner of sounding them are next ably discussed. Here the reader should verify the correctness of many of the statements by experiments on his own sound-producings organs, and thus impart a personal and therefore increased interest to his reading. The chapter on the affections of speech is one of the most interesting in the book, but, from the limited space at our disposal we can do no more than mention it, recommending it specially to our readers, particularly that portion of it treating of "affections arising from defective education and the acquirement of bad habits." In conclusion, Dr. Bristowe gives a few hints on public speaking and singing, which are in keeping with the general excellence of the work.

* I. *The Eye and Sight.* By Henry Power, F.R.C.S.—II. *The Ear and Hearing.* By George P. Field, Aural Surgeon to St. Mary's, etc.—III. *The Throat, Voice, and Speech.* By John S. Bristowe, M.D., F.R.S.—Cassell and Co., London.

